# Looking back: forward looking

**DOI:** 10.1093/gigascience/gix064

**Published:** 2017-07-24

**Authors:** Scott C. Edmunds, Nicole A. Nogoy, Hans Zauner, Peter Li, Christopher I. Hunter, Xiao Si Zhe, Laurie Goodman

**Affiliations:** GigaScience, BGI Hong Kong Ltd., 16 Dai Fu Street, Tai Po Industrial Estate, NT, Hong Kong SAR, China

## Abstract

*GigaScience* is now 5 years old, having been launched at the 2012 Intelligent Systems for Molecular Biology conference. Anyone who has attended what is the largest computational biology conference since then has had the opportunity to join us for each birthday celebration—and receive 1 of our fun T-shirts as a party prize. Since launching, we have pushed our agenda of openness, transparency, reproducibility, and reusability. Here, we look back at our first 5 years and what we have done to forward our open science goals in scientific publishing. Our mainstay has been to create a process that allows the availability and publication of as many “research objects” as possible to create a more complete way of communicating how the research process is done.

Scientific studies are more than just the narrative of the findings; they’re also the data behind the work. With this in mind, in 2010 the leaders of BGI (formerly the Beijing Genomics Institute) conceived the idea for a new model for life-science publishing. As 1 of the world's largest genomics organizations, they realized that with BGI’s huge computational capacity they could provide the means to link standard published articles directly with the data supporting the research. By doing this as part of a publication system, it would be possible for research articles to be more comprehensive and for data producers to finally be able to get credit—via citations—for generating broadly useful datasets. Thus, *GigaScience* was created [1].

From the beginning, our goal was to change the way scientific publishing was done; with reproducibility, reusability, accessibility, transparency, and openness being our core principles. To do this, our first step was to find ways to directly link and make citable all “research objects” (data, software tools, and workflows) that were used or created during research. With the computational and storage resources at BGI, we were easily able to develop a database to host any type or size of data: *Giga*DB (GigaDB, RRID:SCR_004002) [2]. By partnering with the British Library and DataCite, we were then able to give each dataset we host a Digital Object Identifier (DOI), allowing direct citation of the data and linking it to the research article describing or using that data. Pre-empting the findable, accessible, interoperable, and re-usable (FAIR) principles of data management [3], we have also tried to aid data interoperability and re-usability by being the first journal to take submissions in the ISA-TAB metadata format and by providing programmatic access to the data by the provision of an application programming interface (API) to *Giga*DB.

After the launch of *Giga*DB, we went on to develop a Galaxy platform, GigaGalaxy, so that computational tools present in manuscripts could be tested and run in this platform [4]. We also have a GitHub site in case authors have not made their source code available, and if the code is already available, we take snapshots in *Giga*DB to ensure that the version-of-record is always available with the paper. With these tools in place and curation staff to help authors, we have been able to have all of these components of the manuscript available during peer review.

A major step toward improving current scientific publishing mechanisms to communicate research is to take advantage of all currently available technical advances to make all research objects accessible to the community. We envision that the future of publishing—communicating research—is to take advantage of these mechanisms and create a new kind of research “Article,” one that integrates different types of research objects (all the products of the research cycle) into the traditional static paper (Fig. 1).

**Figure 1:**
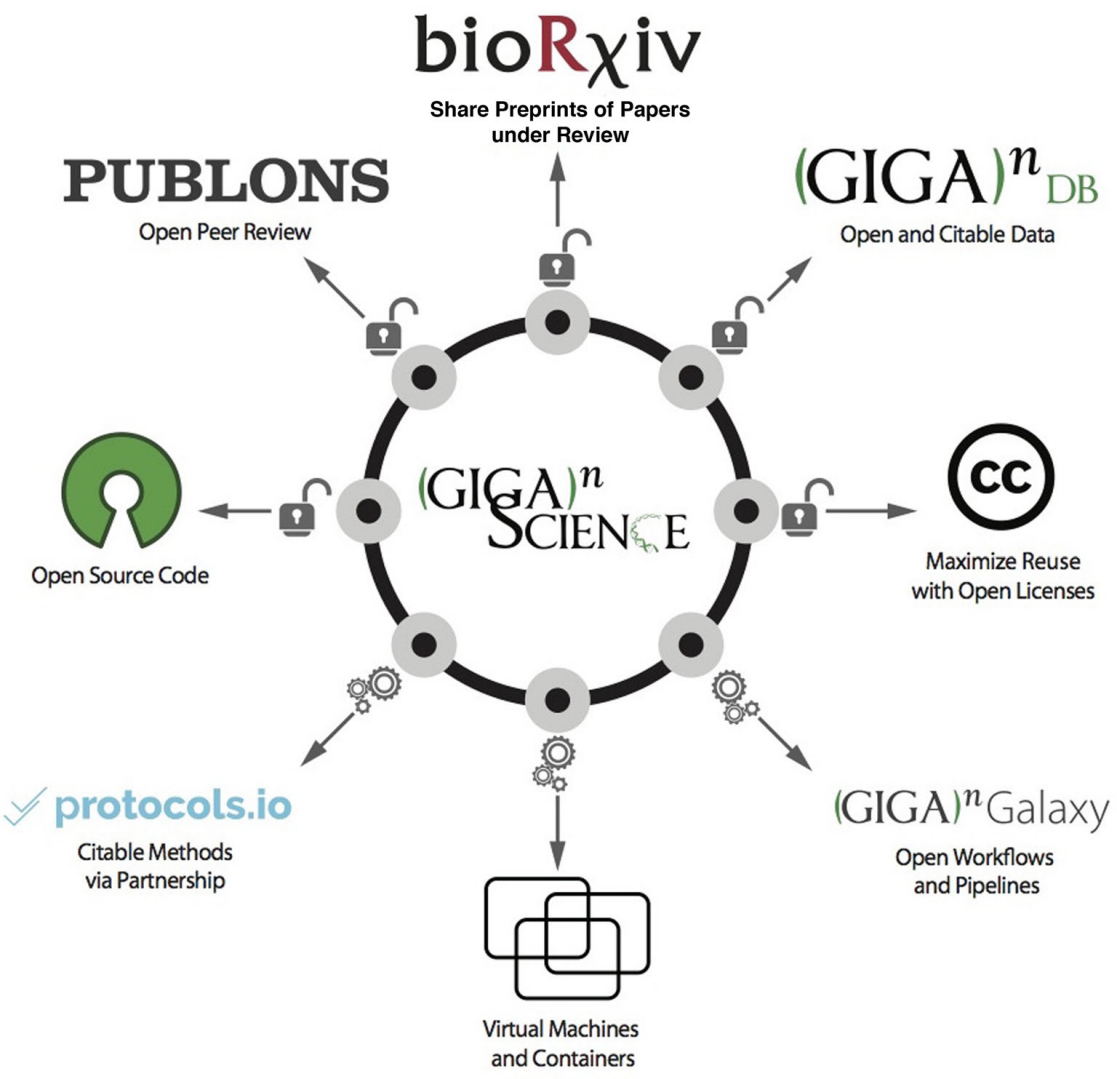
The *GigaScience* principles and practices for publishing and integrating all research objects to maximize reproducibility, transparency, and reuse.

On top of publishing Galaxy workflows, we have published the whole reproducibility toolkit, with packages to recreate papers in Knitr, virtual machine packages of code and data, and a growing number of computational workflows containerized in Docker. Wet protocols are also taken care of through integration with the protocols.io protocol repository. The latest platform we’ve experimented with is Code Ocean [5], where they wrap and encapsulate the data, code, and computation environment in a “Compute Capsule” that can be cited with a DOI or inspected and interacted with through their platform and embedded plugins. We’ve now embedded this into our *Giga*DB entries (see our first example [6]). For more detail and further notable examples of our work in these areas, you can read our published peer-reviewed work on our publication pipelines [7] and reproducibility case studies [8].

Building our own data hosting infrastructure has helped fill many gaps in the reproducibility cycle, but we don’t need to reinvent the wheel—forming partnerships and integrating with protocols and computational workflow repositories and platforms has enabled us to quickly adapt and respond to the dynamic challenges and developments that publishing at the front line of data-driven research entails. Using the unifying factors of open source, open licensing, and APIs, we have managed to build a pipeline connecting platforms, such as bioRxiv, protocols.io, Code Ocean, and Publons—allowing transparent publication of all research objects. Further, we feel that partnerships that enhance the speed of data release are key. Thus, from the first days of launching *GigaScience*, we emphasized that we do not see preprints as a “prior publication” that would preclude submission to us, and we have strongly encouraged preprint server use. In fact, from launch we have been regularly inviting authors of appropriate-looking preprints to submit to the journal. Authors can now submit their bioRxiv preprints directly to us via the biorXiv B2J platform at the push of a button. This technical integration is another hallmark of biology preprints becoming a standard, accepted, and faster way of communicating research results.

Openness from the beginning has been crucial: it is essential for transparency in the publishing industry, but also allows researchers and citizen scientists alike to assess the content of the work, reuse all of the information, understand what goes into assessing a manuscript, and more. We are quite obviously an open access journal, but since research is far more than a PDF, we mandate that the authors *must* make all their data, source code, software, and any other relevant information or material openly and freely available. (Note, however, that we do have special mechanisms for data that needs to be in a secure database.)

It's easy to mandate, but we also recognize the burden this places on authors, who already have enough to do. Given our very strict sharing rules, we try to take the burden off the authors by having procedures and tools to help. Orphan data from the manuscript can be hosted in *Giga*DB, and we have biocurators to help authors migrate and organize their data. Moreover, since there are other databases that are mandatory for use when publishing—in particular, raw sequencing data must go into one of the International Nucleotide Sequence Database Consortium repositories (NCBI, EBI, or DDBJ)—our publication process includes a check at the beginning to make sure the authors have already deposited their data there; if there is a problem, our experienced curators can help authors. Doing such checks at the start is especially good as it can be much harder to get these data released after publication, as many journals have found.

For further transparency, we also mandate open peer review, so reviewers are named. Thus, the guessing games of “the reviewer is out to get me” and the lack of context for understanding review content are simply gone. We have found that the review process is more constructive than antagonistic, and we’ve also had a “meta” (real-time, open) peer review that included blog and twitter discussions of a paper while it was under review. (See more in “Peering into peer review” [9].) Additionally, because peer review is an integral part of the scientific process, it should not be inaccessible to the community, or worse—lost. Therefore, we make the reviews, editor comments, author comments, and all versions of the manuscript available to the public after publication.

While scientific publishing in many areas has been slow to keep up with the pace of research, change was our goal from the beginning. Over the last 5 years, we have worked hard to be as responsive as possible, embraced ongoing and current technologies, and immediately adopted emerging technical tools that clearly helped move research communication in an open, transparent, and rapid direction. We have focused on data and reproducibility, rather than subjective and outdated metrics such as Impact Factor [10], and we’d like to think this approach has paid dividends through the examples of re-use and by seeing more traditional publishers beginning to publish data-centric papers and, like us, working to adopt the FAIR principles for data [3]. The scientific publishing industry still has a long way to go to capture and make available as much of the research process as possible, but we at *GigaScience* will continue to push our agenda of identifying needs and adopting tools and mechanisms that will continue to better link and speed the pace of scientific communication for our next 5 years and beyond.

## Abbreviations

API: application programming interface; DOI: Digital Object Identifier; FAIR principles of data: findable, accessible, interoperable, and re-usable.

## Competing interests

The authors are all employed by *GigaScience*.

## Funding

The journal has been supported by Article Processing Charges and funding from BGI and China National Genebank, as well as by the UK Natural Environmental Research Council (NERC; NE/K011294/1) and BBSRC China Partnering Awards (BB/J020265/1 and BB/M027635/1).

## Author contributions

L.G. and S.C.E. wrote the original draft, and the other authors were involved in review and editing.

## Supplementary Material

GIGA-D-17-00174_Original-Submission.pdf
